# Impact of Autoimmune Gastritis on Occurrence of Metachronous Gastric Neoplasms after Endoscopic Resection for Gastric Neoplasms

**DOI:** 10.3390/cancers15194859

**Published:** 2023-10-05

**Authors:** Donghoon Kang, Chul-Hyun Lim, Jin Su Kim, Yu Kyung Cho, Jae Myung Park, Myung-Gyu Choi

**Affiliations:** Department of Internal Medicine, The Catholic University of Korea College of Medicine, Seoul 06591, Republic of Korea; etiria@catholic.ac.kr (D.K.); jinsu@catholic.ac.kr (J.S.K.); ykcho@catholic.ac.kr (Y.K.C.); parkjerry@catholic.ac.kr (J.M.P.); choim@catholic.ac.kr (M.-G.C.)

**Keywords:** autoimmune gastritis, metachronous gastric neoplasms, endoscopic resection, gastric cancer, anti-parietal cell antibody

## Abstract

**Simple Summary:**

Autoimmune gastritis (AIG), characterized by antibody production against gastric parietal cells, is associated with a higher incidence of neuroendocrine tumors and gastric cancers. Metachronous gastric neoplasms become a major concern after endoscopic resection (ER) for early gastric cancer lesions. We assessed the impact of AIG on MGN following ER. The AIG group had higher MGN rates (45.0% vs. 18.3%), with similar patterns of metachronous tumors. Multivariate analysis revealed AIG (HR 3.32) was linked to MGN occurrence. Because AIG patients face a greater MGN risk post-ER, positive anti-parietal cell antibody test results necessitate vigilant monitoring and management for timely treatment.

**Abstract:**

Gastric cancer is the fifth most common cancer and the third leading cause of cancer-related deaths worldwide. Autoimmune gastritis (AIG) is characterized by antibody production against the gastric parietal cells, reducing the number of functional parietal cells. It is also associated with an increased susceptibility to gastric neuroendocrine tumors and gastric cancer. Endoscopic resection (ER) is an effective treatment for early gastric cancer; however, metachronous gastric neoplasms (MGN) can develop. This study aimed to evaluate the clinical effect of AIG on the occurrence of MGN after ER for gastric neoplasms. We retrospectively analyzed patients who underwent ER for gastric neoplasms. Patients with multiple lesions, recurrent lesions, or a history of partial gastrectomy were excluded. The presence of AIG was determined using anti-parietal cell antibody (APCA) testing. Follow-up endoscopy and metachronous tumor occurrence rates were compared between the AIG and non-AIG groups. Of the 569 patients, 282 underwent APCA testing and 20 (7.1%) were diagnosed with AIG. The incidence of MGN was significantly higher in the AIG group than in the non-AIG group (45.0% vs. 18.3%); however, the MGN occurrence pattern was similar between the two groups. Multivariate analysis revealed that AIG (HR 3.32, 95% CI 1.55–7.10, *p* = 0.002) and a higher body mass index (HR 1.16, 95% CI 1.06–1.27, *p* = 0.002) were independent factors significantly associated with the occurrence of MGN. Patients with AIG have a higher risk of metachronous lesion occurrence after ER for gastric neoplasms. Positive results of APCA testing have independent clinical implications for predicting MGN. Proper monitoring and management are essential for early detection and treatment of recurrent lesions in patients with AIG.

## 1. Introduction

Gastric cancer is the fifth most common cancer and the third leading cause of cancer-related deaths worldwide [1,2]. The risk of developing gastric adenocarcinoma, responsible for over 90% of gastric cancers, is influenced by multiple factors, including *H. pylori* infection, dietary components, and active tobacco smoking, with *H. pylori* infection being the predominant association [3]. A consistent and sustained decline in the incidence of gastric cancer was observed globally between 1988 and 2012. Several factors likely contributed to this trend, including reduced smoking rates, decreased prevalence of *H. pylori* infection, and improved dietary patterns [4].

Autoimmune gastritis (AIG) typically manifests as a corpus-restricted mucosal atrophy and arises from an autoimmune process characterized by the production of antibodies targeting gastric parietal cells, the so-called anti-parietal cell antibodies (APCAs), thereby resulting in a reduction in the quantity of functional parietal cells [5,6,7]. The clinical presentation of AIG can range from asymptomatic to symptomatic, manifesting as pernicious or iron-deficiency anemia. Although the precise etiology of AIG remains unclear, studies have shown a correlation between AIG and elevated susceptibility to gastric neuroendocrine tumors as well as gastric cancer [8]. In a Danish study, the calculated incidence of gastric cancer in patients with pernicious anemia was three times higher than that in the general population [9]. One of the most commonly known mechanisms of gastric cancer development is Correa’s cascade [10]. Prolonged exposure to chronic inflammation in the stomach leads to gastric carcinogenesis, progressing through stages of gastritis, atrophy, metaplasia, dysplasia, and carcinoma. In AIG patients, profound and continuous exposure to gastrin might play a role in gastric carcinogenesis through chronic inflammation [11]. The matter of whether individuals with AIG should undergo routine upper endoscopic surveillance to assess their cancer risk has been a subject of deliberation.

Endoscopic resection (ER), including endoscopic mucosal resection (EMR) and endoscopic submucosal dissection (ESD), has gained widespread acceptance as an effective treatment modality for early gastric cancer (EGC) with a low risk of lymph node metastasis [12]. Current guidelines also recommend the removal of adenomas along with EGCs for diagnostic and therapeutic purposes based on the fact that adenomas are considered a precancerous lesion [12,13]. However, because ER preserves the stomach, metachronous gastric neoplasms (MGN) can develop.

Gastric cancer is characterized by the accumulation of genetic and epigenetic alterations. Aberrant DNA methylation has recently emerged as an important mechanism in gastric carcinogenesis. Chronic inflammation leads to increased cytokine secretion and hypermethylation of the promoter regions of tumor suppressor genes. Consequently, the cumulative inactivation of tumor suppressor genes ultimately results in the development of gastric cancer. This phenomenon is known as field cancerization, in which premalignant lesions may already exist in areas of the stomach even before visible signs of gastric cancer appear. Therefore, it is reasonable to hypothesize that higher levels of aberrant DNA methylation related to gastric carcinogenesis in patients undergoing ER for gastric neoplasms may indicate a higher risk of MGN due to field cancerization.

The development of high-risk metaplastic lesions within the gastric mucosa occurs in both autoimmune- and infection-induced gastritis, thereby raising concerns regarding the potential for carcinogenesis under both inflammatory conditions [11]. Therefore, in this study, we aimed to assess the prevalence of AIG in patients with gastric neoplasms who underwent ER and investigate the effect of AIG on the occurrence of MGN.

## 2. Materials and Methods

### 2.1. Patients

**Figure 1:**
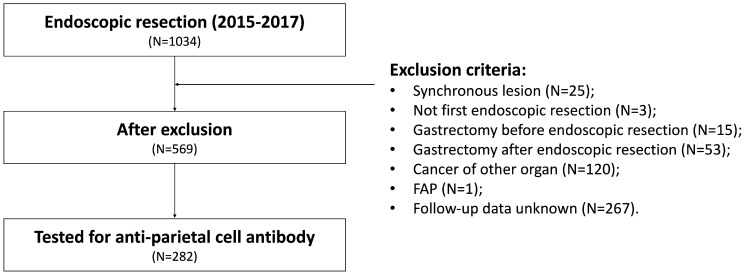
Study flowchart. FAP: familial adenomatous polyposis.

A retrospective analysis was conducted on patients who underwent ER for gastric neoplasia at Seoul St. Mary’s Hospital in Seoul, Korea, between January 2015 and December 2017. ER was performed when the lesion met the indication of the Japan Gastric Cancer Association (JGCA) guideline for gastric cancer treatment [14]. We analyzed the medical records of these patients until December 2022 to obtain follow-up data for more than 5 years. To reduce potential bias in the clinical outcomes of ER, patients with multiple lesions, recurrent lesions at a prior resection site, or metachronous lesions were excluded. Patients with a remnant stomach who had previously undergone partial gastrectomy for any reason were also excluded from the study. A total of 1034 patients underwent ER for gastric neoplasms during the study period. Patients with multiple lesions (n = 25), metachronous lesions (n = 3), or a history of partial gastrectomy (n = 15) were excluded. Patients who underwent gastrectomy after ER (n = 53), those with cancer in other organs (n = 120), those with familial adenomatous polyposis (n = 1), and those who lacked follow-up data (n = 267) were also excluded to focus on long-term outcomes during the follow-up period. The remaining 569 patients were included in the analysis. Among these patients, those who underwent APCA testing at the endoscopic resection (n = 282) were finally included in the analysis (Figure 1). A subgroup of patients who tested positive for APCA was designated the AIG group. In the APCA test, patient serum was processed using reagents (Mosaic Basic profile 2; Euroimmune, Lübeck, Germany) and antibodies were subsequently identified through immunofluorescence methods. Cases were classified as negative if the titer was below 1:40.

### 2.2. Endoscopic Procedures and Follow-Up

Indigo carmine solution was sprayed onto the gastric mucosa; lesion marking and ER, including EMR or ESD, were performed using a hook knife and snare (Olympus Medical Systems Co. Ltd., Tokyo, Japan). Electronic coagulation was performed simultaneously during resection. All ESD procedures were performed by expert endoscopists with more than 5 years of experience. The extent of atrophic gastritis was classified as closed- or open-type based on the Kimura–Takemoto classification [15]. *H. pylori* infection was diagnosed based on one of the following positive results: rapid urease test (CLO test; Ballard Medical Products, Draper, UT, USA), polymerase chain reaction (PCR) test, histological examination using Warthin–Starry silver staining, and serum *H. pylori* antibody at the time of ER of gastric neoplasia.

After ER of gastric neoplasia, all patients were scheduled for a follow-up endoscopy at 3, 6, and 12 months, and annually thereafter. Biopsies were obtained for all suspicious mucosal lesions and evaluated histologically during the follow-up endoscopic examination. A surveillance endoscopy was performed by the same endoscopist who performed the initial ESD.

### 2.3. Clinicopathological Evaluations

We evaluated the clinical characteristics and histopathology of the three types of lesions. The tumor location along the longitudinal axis of the stomach was determined by dividing it into three equal sections: upper, middle, and lower. The final histological reports of all the lesions were documented following the JGCA guideline [14]. Patients with *H. pylori* infection were categorized into four groups: *H. pylori* negative (HPN), *H. pylori* eradicated (HPE), *H. pylori* persistent (HPP), and *H. pylori* status unknown (HPU). HPN indicated patients with consistently negative *H. pylori* test results throughout the follow-up period. HPE referred to patients who initially tested positive for *H. pylori*, received eradication therapy, and achieved successful eradication. HPP indicated patients who tested positive for *H. pylori* but did not undergo eradication therapy. HPU represented the cases in which *H. pylori* test results were not obtained during the study period.

### 2.4. Outcome Measures

The primary outcome was the clinical effects of AIG on the occurrence of MGN after ER for gastric neoplasms. The secondary outcome was the prevalence of AIG in regions with a high incidence of gastric cancer and its effect on the occurrence of MGN after ER for gastric adenocarcinoma lesions.

### 2.5. Statistical Analyses

Continuous data are presented as the mean ± standard deviation and categorical data are presented as quantities and proportions. A comparison of baseline characteristics of the AIG group was performed using the chi-square test for nominal variables and Student *t* test or Mann–Whitney U test for numeric values. The cumulative incidence of gastric cancer and neoplasia after ESD was analyzed using the Kaplan–Meier method and the differences between the two groups were compared using the log-rank test. A Cox proportional hazards model was used to analyze the independent association between AIG and the development of metachronous tumors. Statistical analysis was performed using R statistical software (v4.1.2; R Core Team 2021) and statistical significance was set at *p* < 0.05.

## 3. Results

### 3.1. Baseline Characteristics

The average age of the patients was 65.1 years, and 201 (71.3%) were males. Of the total number of patients, 20 (7.1%) had AIG. The AIG group exhibited a significantly higher incidence of MGN than the non-AIG group (45.0% vs. 18.3%, *p* = 0.010). Factors such as age, sex, pathology of the primary resected lesion, *H. pylori* status, size and location of the initial lesion, and mucosal atrophy did not differ significantly. A summary of baseline characteristics is presented in Table 1.

### 3.2. Metachronous Tumor Occurrence Patterns in Both Groups

**Figure 2:**
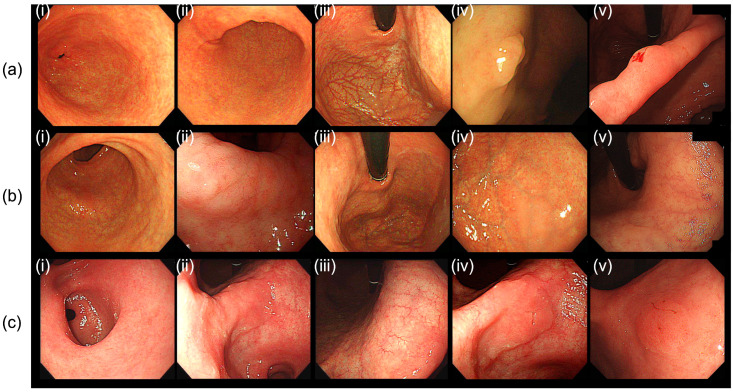
Endoscopic images of a patient with autoimmune gastritis (AIG) with the initial presence of resected gastric neoplasms and metachronous gastric neoplasms: (**a**) Case 1: 52-year-old male. Initial lesion was adenoma. Metachronous lesion with adenoma was observed after 30 months from the initial endoscopic resection; (**b**) Case 2: 71-year-old female. Initial lesion was tubular adenocarcinoma, well-differentiated. Metachronous lesion with adenoma was observed after 41 months from the initial endoscopic resection (**c**) Case 3: 82-year-old male. Initial lesion was tubular adenocarcinoma, well-differentiated. Metachronous lesion with adenoma was observed after 26 months from the initial endoscopic resection. (**i**) Endoscopic image of antrum; (**ii**) Angularis; (**iii**) Corpus; (**iv**) Initial lesion; (**v**); Metachronous tumor lesion.

Table 2 presents the patterns of the metachronous lesions. Although the occurrence rate of metachronous lesions differed significantly between the non-AIG and AIG groups, the occurrence patterns were similar. Approximately 50% of the initial neoplastic lesions were identified as carcinomas in both groups (44.4% and 45.8% in the AIG and non-AIG groups, respectively). Among patients with AIG, 22.2% of the metachronous lesions were diagnosed as carcinomas, whereas in the non-AIG group, 35.4% of the metachronous lesions were classified as carcinomas. Three cases of endoscopic images of the antrum, angularis, corpus, initial lesion, and MGN lesions occurring in AIG patients are shown in Figure 2.

**Figure 3:**
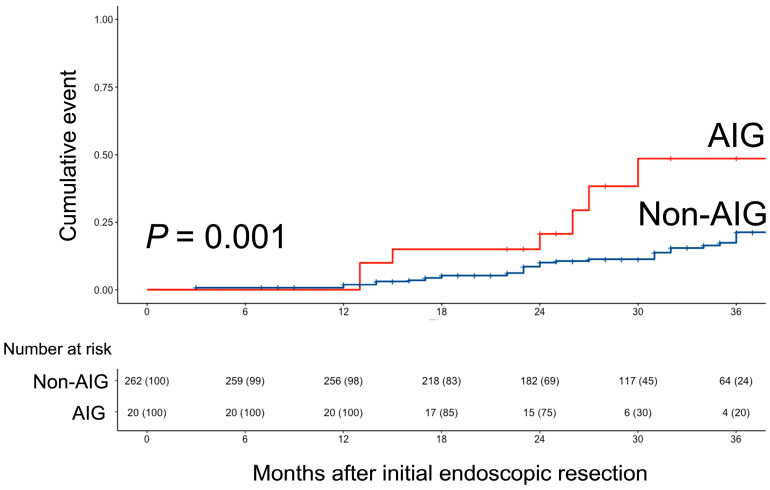
Comparison of the risk of MGN occurrence after endoscopic resection for gastric neoplasm between the AIG and Non-AIG group. AIG, autoimmune gastritis.

As illustrated in Figure 3, the patients in the AIG group exhibited a higher incidence of metachronous occurrence than those in the non-AIG group (*p* = 0.001).

In the univariate analysis, several factors were found to be associated with the occurrence of MGN. These included age (HR 1.03, 95% CI 1.00–1.06, *p* = 0.043), body mass index (BMI: HR 1.16, 95% CI 1.06–1.28, *p* = 0.001), pathology primarily indicating cancer compared to adenoma (HR 0.57, 95% CI 0.34–0.97, *p* = 0.038), and the presence of AIG (HR 3.16, 95% CI 1.53–6.52, *p* = 0.002). After controlling for potential confounding factors, the multivariate analysis revealed that BMI (HR 3.32, 95% CI 1.55–7.10, *p* = 0.002) and AIG (HR 3.32, 95% CI 1.55–7.10, *p* = 0.002) were the only independent factors significantly associated with the occurrence of MGN. The results are summarized in Table 3.

### 3.3. Subgroup Analysis Based on the Initial Pathology

**Figure 4:**
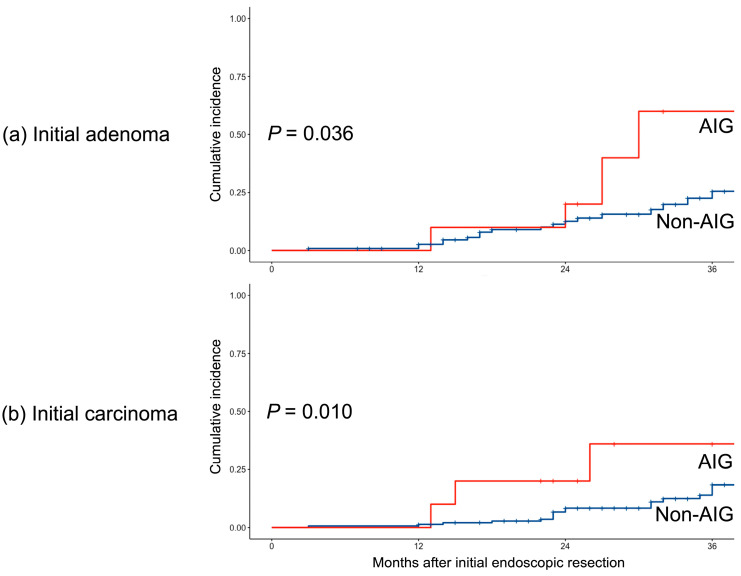
Subgroup analysis of the risk of MGN occurrence in two different pathologies at the initial endoscopic resection. MGN, metachronous gastric neoplasms; AIG, autoimmune gastritis.

Subgroup analyses were performed based on the initial pathology of the ER specimens. In both the initial adenoma and carcinoma groups, the occurrence rates were higher in patients with AIG (*p* = 0.036 for adenoma and *p* = 0.010 for carcinoma) (Figure 4).

## 4. Discussion

In our study, the AIG group showed a more than three-fold higher incidence of MGN occurrence than the non-AIG group. There was no significant difference in the presence of adenoma or cancer during the initial ER between the AIG and non-AIG groups. Furthermore, there were no significant differences in the characteristics of the lesions in MGN. This suggests that there are no substantial differences in the nature of recurrent tumors between the AIG and the non-AIG groups. Therefore, these results emphasize the clinical significance of positive APCA results used for the diagnosis of AIG, as they have an independent impact on the occurrence of MGN.

Previous studies reported no difference among the factors commonly known to be major contributors to the occurrence of MGN, such as the degree of atrophy or eradication of *H. pylori*, between the two groups [16,17,18,19]. On the contrary, an increase in BMI was found to significantly contribute to the occurrence of MGN in our study. In our study, a significant proportion (80%) of the participants exhibited advanced baseline mucosal atrophy, specifically the open type. This finding differs from that of a previous study that suggested a protective effect of *H. pylori* eradication in preventing the occurrence of gastric neoplasms [19], where a smaller proportion of marked mucosal atrophy was observed (70%) in the participants. Consequently, our finding that the extent of mucosal atrophy was not independently associated with the occurrence of MGN can be explained by the presence of a “point-of-no-return” in the development of these lesions.

Although there have been reports associating autoimmunity with the development of gastric cancer through various pathways [20,21], the specific direct mechanisms involved in AIG remain unclear. Further studies are necessary to investigate the contributions of these mechanisms in the development of AIG and its associated etiology.

A recent study conducted in Italy focused on “pure AIG” by selecting only *H. pylori* naïve patients [22]. The findings revealed consistent inflammation primarily affecting the oxyntic region of the stomach. Interestingly, there was no significant increase in gastric cancer risk observed during the follow-up period. The researchers proposed that the elevated risk of gastric cancer observed in AIG patients could be attributed to unrecognized or concurrent *H. pylori* infection. Given the high prevalence of *H. pylori* infection in Korea, it is possible that this factor may explain the higher rate of MGN in AIG patients in our study results.

To the best of our knowledge, this is the first study to evaluate the effect of AIG on the occurrence of MGN. ER of the aforementioned tumor lesions has a positive effect on improving the quality of life of patients before surgery. Patients with AIG need vigilant monitoring and management due to the potential for MGN occurrence, necessitating appropriate follow-up measures.

However, our study had some limitations. First, the number of patients with metachronous occurrence in the AIG group was relatively small. A larger number of patients with metachronous occurrence in the AIG group would have been beneficial for a more in-depth analysis. Nonetheless, despite the small sample size, the statistical power of our study exceeded 0.80, which is considered sufficient. Second, the enrolled patients were solely of Korean ethnicity. Therefore, the results of our study may not be generalizable to other ethnic groups. Variations in genetic and environmental factors among different ethnicities may influence the outcomes. Third, gastrin levels were not analyzed. It is believed that elevated gastrin levels contribute to inflammation in autoimmune gastritis, leading to the development of adenocarcinoma and gastric neuroendocrine tumor I. However, in our study, we examined the serum gastrin levels at the time of resection of gastric epithelial neoplasms. Unfortunately, the measurement of gastrin levels was only available for a subset of patients, and some of them were also taking medications, including proton pump inhibitors, which made it impossible to analyze. As a result, serum gastrin levels were excluded from the primary analysis. Instead, we assessed the extent of mucosal inflammation and atrophy through endoscopic findings. Although serum gastrin levels could provide additional insights, our focus on mucosal atrophy as assessed via endoscopy still yielded valuable information.

Our analysis revealed noteworthy findings regarding the incidence and characteristics of MGN in patients with AIG. By examining the long-term follow-up data, we were able to establish a clearer understanding of the temporal development and impact of MGN in this specific patient population. Furthermore, our findings were statistically significant which provided robust evidence to support our conclusions. Most importantly, there is a significant advantage in providing a practical message that measuring APCA in patients undergoing ER can help predict their prognosis.

Our study highlights the importance of AIG in predicting the occurrence of MGN after ER for gastric tumor lesions. These findings suggest that evaluating the APCA status and implementing appropriate surveillance strategies can aid in identifying high-risk patients and optimizing their management. Further research with larger cohorts and a prospective design is warranted to validate our findings.

## 5. Conclusions

Patients with AIG are at an increased risk of developing MGN following ER. The positive results of APCA testing independently hold clinical significance for predicting MGN. Given these findings, a more vigilant follow-up strategy is warranted for AIG patients after ER for gastric neoplasms.

## Figures and Tables

**Table 1:** Basal characteristics of the patients.

	Total (N = 282)	Non-AIG (N = 262)	AIG (N = 20)	*p* Value
Age (mean ± SD)	65.1 ± 9.4	65.1 ± 9.3	65.0 ± 11.8	0.965
Male, n (%)	201 (71.3%)	187 (71.4%)	14 (70.0%)	1.000
BMI, kg/m^2^, (mean ± SD)	24.2 ± 3.1	24.2 ± 2.9	22.9 ± 5.4	0.296
Cardiovascular disease, n (%)	5 (1.8%)	5 (2.0%)	0 (0.0%)	1.000
Cerebrovascular disease, n (%)	2 (0.7%)	2 (0.8%)	0 (0.0%)	1.000
Diabetes mellitus, n (%)	48 (17.5%)	44 (17.3%)	4 (21.1%)	0.915
Hypertension, n (%)	104 (38.0%)	99 (38.8%)	5 (26.3%)	0.402
Initial carcinoma, n (%)	156 (55.3%)	146 (55.7%)	10 (50.0%)	0.792
Pathologic diagnosis, n (%)				0.670
Tubular adenocarcinoma, WD	116 (41.2%)	110 (42.0%)	6 (30.0%)	
Tubular adenocarcinoma, MD	32 (11.3%)	30 (11.5%)	2 (10.0%)	
Poorly differentiated adenocarcinoma	7 (2.5%)	6 (2.3%)	1 (5.0%)	
Adenoma	127 (45.0%)	116 (44.3%)	11 (55.0%)	
*H. pylori* status, n (%)				
Negative	136 (48.2%)	124 (47.3%)	12 (60.0%)	0.750
Eradicated	111 (39.4%)	105 (40.1%)	6 (30.0%)	
Persistent	16 (5.7%)	15 (5.7%)	1 (5.0%)	
Unknown	19 (6.7%)	18 (6.9%)	1 (5.0%)	
Lesion size, mm (mean ± SD)	15.3 ± 11.7	15.3 ± 11.7	16.1 ± 12.0	0.749
Location, n (%)				
Lower	162 (57.4%)	150 (57.3%)	12 (60.0%)	0.830
Middle	99 (35.1%)	93 (35.5%)	6 (30.0%)	
Upper	21 (7.4%)	19 (7.3%)	2 (10.0%)	
Mucosal atrophy				
No atrophy	2 (0.7%)	2 (0.8%)	0 (0.0%)	0.379
Closed type	43 (15.2%)	42 (16.0%)	1 (5.0%)	
Open type	237 (84.0%)	218 (83.2%)	19 (95.0%)	
MGN occurrence, n (%)	57 (20.2%)	48 (18.3%)	9 (45.0%)	0.010
MGN pattern, n (%)				
No MGN	225 (79.8%)	214 (81.7%)	11 (55.0%)	0.048
Adenoma → Adenoma	23 (8.2%)	19 (7.3%)	4 (20.0%)	
Adenoma → Carcinoma	8 (2.8%)	7 (2.7%)	1 (5.0%)	
Carcinoma → Adenoma	15 (5.3%)	12 (4.6%)	3 (15.0%)	
Carcinoma → Carcinoma	11 (3.95)	10 (3.8%)	1 (5.0%)	

AIG, autoimmune gastritis; BMI, body mass index; SD, standard deviation; WD, well-differentiated; MD, moderately differentiated; MGN, metachronous gastric neoplasm.

**Table 2:** Comparison of the metachronous tumor occurrence patterns between the AIG and non-AIG groups.

N = 57	Non-AIG (N = 48)	AIG (N = 9)	*p* Value
Adenoma → Adenoma	19 (39.6%)	4 (44.4%)	0.878
Adenoma → Carcinoma	7 (14.6%)	1 (11.1%)	
Carcinoma → Adenoma	12 (25.0%)	3 (33.3%)	
Carcinoma → Carcinoma	10 (20.8%)	1 (11.1%)	

AIG, autoimmune gastritis.

**Table 3:** Univariate and multivariate analyses of the MGN occurrence.

N = 282	Overall Survival			
	Univariate Analysis		Multivariate Analysis	
	HR (95% CI)	*p* Value	HR (95% CI)	*p* Value
Age	1.03 (1.00–1.06)	0.043	1.03 (1.00–1.06)	0.070
Male sex	1.21 (0.66–2.21)	0.500	0.97 (0.51–1.87)	>0.900
BMI	1.16 (1.06–1.28)	0.001	1.16 (1.06–1.27)	0.002
Primarily cancer	0.57 (0.34–0.97)	0.038		
AIG	3.16 (1.53–6.52)	0.002	3.32 (1.55–7.10)	0.002
*H. pylori* status				
HPE (vs. HPN)	0.70 (0.38–1.27)	0.200	0.85 (0.45–1.60)	0.600
HPP (vs. HPN)	0.94 (0.29–3.09)	>0.900	0.85 (0.25–2.90)	0.800
HPU (vs. HPN)	0.45 (0.11–1.89)	0.300	0.43 (0.10–1.85)	0.300
Mucosal atrophy (closed vs. open)	1.03 (0.52–2.04)	>0.900	1.01 (0.49–2.07)	>0.900
Initial lesion location				
Middle (vs. Lower)	0.92 (0.52–1.61)	0.800		
Upper (vs. Lower)	0.98 (0.35–2.79)	>0.900		

MGN, metachronous gastric neoplasm; HR, hazard ratio; CI, Confidence interval; HPE, *H. pylori* eradicated; HPN, *H. pylori* negative; HPP, *H. pylori* persistent; HPU, *H.pylori* status unknown.

## Data Availability

The data presented in this study are available on request from the corresponding author. The data are not publicly available due to ethical restrictions.
